# United States Civil Service Examination

**Published:** 1917-01

**Authors:** 


### United States Civil Service Examination.

The United States Civil Service Commission announces an open competitive examination for assistant epidemiologist, for men only. From the register of eligibles resulting from this examination certification will be made to fill vacancies in this position in the Public Health Service, at salaries ranging from $2000

to $2500 a year, and vacancies as they may occur in positions requiring similar qualifications, unless it is found to be in the interest of the service to fill any vacancy by reinstatement, transfer, or promotion. Certification to fill the higher salaried positions will be made only from those attaining the highest average percentages in the examination.

The duties of this position will be to make epidemiologic and sanitary surveys to determine the prevalence and causation of disease, to conduct laboratory studies in relation thereto, and to recommend measures to prevent and control outbreaks of disease.

It is desired to secure persons thoroughly competent in the various branches of sanitary bacteriology, and especially in isolating the typhoid bacillus from infected persons and materials.

Competitors will not be required to report for examination at any place, but will be rated on the following subjects, which will have the relative weights indicated:

| Subjects. | Weights. |
|---|---|
| 1. General education and medical training | 25 |
| 2. Laboratory experience | 25 |
| 3. Experience in epidemiological work | 40 |
| 4. Publications or thesis | 10 |
| Total | 100 |

Graduation from a medical school of recognized standing, and at least three years' experience in epidemiological work under Federal, State, or local authorities, and experience in laboratory technique, especially in regard to malaria and typhoid fever, are prerequisites for consideration for this position.

If a thesis is submitted under subject 4 it must be on some sanitary subject upon which the candidate has done special work.

Statements as to education and experience are accepted subject to verification.

Applicants must have reached their twenty-third but not their fortieth birthday on the date of the examination.

This examination is open to all men who are citizens of the United States and who meet the requirements.

Persons who meet the requirements and desire this examination should at once apply for Form 304 and special form, stating the title of the examination desired, to the United States Civil Service Commission, Washington, D. C.; the Secretary of the United States Civil Service Board, postoffice, Boston, Mass.;

Philadelphia, Pa.; Atlanta, Ga.; Cincinnati, Ohio; Chicago, Ill.; St. Paul, Minn.; Seattle, Wash.; San Francisco, Cal.; customhouse, New York, N. Y.; New Orleans, L. A.; Honolulu, Hawaii; old customhouse, St. Louis, Mo.; administration building, Balboa Heights, Canal Zone; or to the Chairman of the Porto Rican Civil Service Commission, San Juan, Porto Rico. Applications should be properly executed, excluding the medical and county officer's certificates, and must be filed with the Commission at Washington, with the material required, prior to the hour of closing business on January 30, 1917.

---

Do We Sleep too Much?—I doubt if many sleep too much. It is possible to encourage a habit of somnolency to the detriment of mind and body. I believe many more worry themselves because they suppose they sleep too little and that if they do not get more sleep their brains will suffer. A needless dread. Many a man will do day by day a long day's work on, say, four or five hours' sleep. It is much a matter of habit with the adult. I remember that Lord Palmerston is supposed to have been able to get through his busy political life by having the knack of dropping off to sleep for a few moments at all sorts of odd times and chances, and there must be many who know well what an enormous restorative value such a lapse may have. Rest should be regular and punctually allotted, and let sleep come during that hour as it may.—Sir James F. Goodhart, Bart., M. D., C. M., F. R. C. P. Lond., in *The Strand.*

---

"Do you see that strong, healthy looking man over there?"

"I was just admiring his physique."

"The doctors gave him up years ago."

"You surprise me."

"Yes. They found they couldn't get anything out of him."—*Birmingham Age-Herald.*

---

A lawyer was arguing with a physician over the relative merits of their respective professions.

"I don't say that all lawyers are villains," said the doctor, "but you'll have to admit that your profession doesn't make angels of men."

"No," retorted the lawyer, "you doctors certainly have the best of us there."—*San Patricio County News.*

